# Psychische Belastung von Kindern und Jugendlichen in der Coronazeit: Literaturüberblick und Projektskizze

**DOI:** 10.1007/s11553-022-00946-0

**Published:** 2022-04-29

**Authors:** Christin Bohl, Pania Karnaki, Simone Cheli, Gertrudis Fornes Romero, Renata Glavak Tkalić, Eva Papadopoulos, Mathieu Schaefer, Hendrik Berth

**Affiliations:** 1 Medizinische Fakultät Carl Gustav Carus, Psychosoziale Medizin und Entwicklungsneurowissenschaften, Forschungsgruppe Angewandte Medizinische Psychologie und Medizinische Soziologie, Technische Universität Dresden, 01307 Dresden, Deutschland; 2 Astiki Mikerdoskopiki Etaireia Prolepsis, 15125 Marousi, Athen, Griechenland; 3 Associazione Tages Onlus, 50137 Florenz, Italien; 4 Polibienestar Research Institute, Universitat de Valencia, 46010 Valencia, Spanien; 5 Institut Drustvenih Znanosti Ivo Pilar, 10000 Zagreb, Kroatien; 6 CSI Center For Social Innovation Ltd, 1010 Nikosia, Zypern; 7 OSENGO, 63000 Clermont-Ferrand, Frankreich

**Keywords:** COVID-19, Psychische Situation, Verhaltensauffälligkeiten, Risikofaktoren, Schulungsprogramm, COVID-19, Mental Situation, Behavioral problems, Risk factors, Training program

## Abstract

**Hintergrund:**

Internationale Studien zeigen, dass die Maßnahmen zur Eindämmung der COVID-19-Pandemie verstärkt zu psychischen Auffälligkeiten bei Kindern und Jugendlichen führen können. Insgesamt gibt es zu diesem Themenbereich viele Studienaktivitäten, jedoch nur wenige repräsentative Untersuchungen für Deutschland.

**Ziel der Arbeit:**

Es soll untersucht werden, welchen Einfluss die COVID-19-Pandemie auf die psychische Gesundheit von Kindern und Jugendlichen in Deutschland nimmt und welche Risiko- und protektive Faktoren für das mentale Wohlbefinden während der Pandemie existieren. Im Anschluss wird ein EU-weites Projekt skizziert, welches die psychische Gesundheit von SchülerInnen durch ein Schulungsprogramm für Lehrkräfte und andere PädagogInnen fördern will.

**Methoden:**

Unter Nutzung der Literaturdatenbanken PubMed und Medline fand eine unsystematische Literaturrecherche im Sinne eines narrativen Reviews statt. Die in dieser Arbeit einbezogenen Studien wurden anhand ihres thematisch passenden Abstracts ausgewählt.

**Ergebnisse:**

Die Zahl der Kinder, die psychische Auffälligkeiten oder Verhaltensstörungen zeigen, stieg rapide an. Vor allem die soziale Isolation, Ängste und Unsicherheit, sowie Konflikte innerhalb der Familie aufgrund von Überforderung oder finanziellen Sorgen führen zu einer Verschlechterung der psychischen Situation von Kindern und Jugendlichen. Die Folgen sind depressive Verstimmungen, Verhaltensauffälligkeiten und psychosomatische Beschwerden.

**Schlussfolgerung:**

Die Auswirkungen der COVID-19-Pandemie auf die psychische Gesundheit von Kindern und Jugendlichen dürfen nicht unterschätzt werden. Auch in den kommenden Jahren ist ein besonderer Unterstützungsbedarf gefordert.

## Abstract (en)

**Background:**

International studies show that measures to contain the COVID-19 pandemic can lead to increased mental health problems in children and adolescents. Overall, there are many study activities on this topic, but only a few representative studies for Germany.

**Objectives:**

The impact of the COVID-19 pandemic on the mental health of children and adolescents in Germany will be investigated, as well as the risk and protective factors for mental well-being during the pandemic. Subsequently, an EU-wide project will be outlined, which aims to promote the mental health of students through a training program for teachers and other educators.

**Methods:**

Using literature databases PubMed and Medline, an unsystematic literature search was carried out in the sense of a narrative review. The studies included in this work were selected based on their thematically appropriate abstract.

**Results:**

The number of children showing mental or behavioral problems increased rapidly. Above all, social isolation, fears and insecurity, as well as conflicts within the family due to excessive demands or financial worries, lead to a deterioration in the psychological situation of children and young people. The consequences are depressive moods, behavioral problems and psychosomatic complaints.

**Conclusion:**

The impact of the COVID-19 pandemic on the mental health of children and adolescents should not be underestimated. There will also be a special need for support in the coming years.

## Hintergrund und Fragestellung

Die COVID-19-Pandemie hat einen internationalen Gesundheitsnotstand mit nie da gewesenem Ausmaß zur Folge. Um diese Krise der öffentlichen Gesundheit schnellstmöglich einzudämmen, wurden Maßnahmen ergriffen, die eine weitere Ausbreitung des Coronavirus verhindern sollten. Diese Maßnahmen waren z. T. umstritten und führten zu deutlichen Einschränkungen des gesellschaftlichen Lebens. Vor allem die geforderte soziale Distanzierung, einschließlich der Schließungen von Kitas, Schulen und Freizeiteinrichtungen, führte zu einschneidenden Veränderungen des Alltags von vielen Kindern und Jugendlichen. Viele Familien fühlten sich bei dem Versuch, Homeoffice und Homeschooling zu vereinbaren, überfordert oder waren von finanziellen Sorgen belastet. Dies führte häufig zu familiären Spannungen und Konflikten [20]. Die Zahl der Kinder, die psychische Auffälligkeiten oder Verhaltensstörungen zeigen, stieg rapide an. Therapieplätze sind rar und mit langen Wartezeiten verbunden [27, 28, 32].

Im Folgenden soll die Fragestellung geklärt werden, welche Auswirkungen die Coronapandemie auf die mentale Gesundheit von Kindern und Jugendlichen in Deutschland hat. Weiterhin sollen mögliche Risiko- und protektive Faktoren analysiert und deren Einfluss auf die psychische Gesundheit von Kindern und Jugendlichen während der COVID-19-Pandemie beschrieben werden. Dazu erfolgte eine unsystematische Literaturrecherche in den Datenbanken PubMed und Medline nach Studien, die sich mit der psychischen Gesundheit von Kindern und Jugendlichen während der Coronapandemie befassten. Bei der Recherche wurden folgende Suchworte verwendet: „Corona“, „children and adolescents“, „psychological distress“ und „behavioral problems“. Es wurden für diese Arbeit 18 Studien anhand ihres thematisch passenden Abstracts ausgewählt. Die Ergebnisse werden im Sinne eines narrativen Reviews dargestellt (Tab. 1). Es wird deutlich, dass v. a. die langfristigen Auswirkungen nicht unterschätzt werden dürfen und es besonderen Unterstützungsbedarf gibt. Die Studienergebnisse flossen in die Entwicklung eines EU-weiten Projekts ein, das abschließend skizziert wird. Damit schließt diese Arbeit eine große Forschungslücke, da bisher kaum Übersichtsarbeiten zu diesem Thema in Deutschland veröffentlicht wurden.

**Tab. 1:** Übersicht der empirischen Befragungen zur mentalen Gesundheit von Kindern und Jugendlichen

Studie	*n*	Alter (Jahre)	Mittleres Alter (Jahre)	Geschlechterverteilung	Instrumente
BELLA-Studie im Kinder- und Jugendgesundheitssurvey (KiGGS) 2007 [26]	2863 Familien	7–17	k. A.	48,5 % weiblich 51,5 % männlich	*Validierte Fragebögen:* SDQ [18]
Calvano et al. 2021 [5]	1024 Familien	< 18	k. A.	k. A.	Computergestützte Telefoninterviews
COPSY-Studie 2021 [27]	1586 Familien mit Kindern im Alter von 7 bis 17 Jahre *n* = 1040 Kinder von 11 bis 17 Jahre	7–17	M = 12,25 (SD = 3,30)	50 % weiblich 50 % männlich	*Validierte Fragebögen:* Psychosomatische Beschwerden [21] KIDSCREEN-10-Index [25] SDQ [13] SCARED [2] CES‑D [1]
DAK-Längsschnittstudie 2019 [10]	1000	12–17	k. A.	k. A.	Computergestützte Telefoninterviews
DAK-Längsschnittstudie 2020 [11]	824 Elternteile und jeweils ein Kind	10–18	k. A.	k. A.	Online-Befragungen
KiGGS-Studie 2018 [19]	KiGGS-Baseline *n* = 14.477 KiGGS-Welle 2 *n* = 13.205	3‑17	k. A.	*KiGGS-Baseline*: 49 % weiblich 51 % männlich *KiGGS-Welle 2:* 50,26 % weiblich 49,74 % männlich	*Validierte Fragebögen:* SDQ [18]
„Kind sein in Zeiten von Corona“ 2020 [20]	*n* = 12.628 Familien *n* = 12.555	3–15	k. A.	49 % weiblich 51 % männlich	Online-Befragungen Interviews
Pieh et al. 2020 [23]	*n* = 3052	14–20	M = 16,47 (SD = 1,45)	70,1 % weiblich 29,9 % männlich	*Validierte Fragebögen:* PHQ‑9 [30] GAD‑7 [22] ISI [12] EAT‑8 [29] WHO‑5 [4]

*n* bezieht sich auf die Anzahl der teilnehmenden Kinder und Jugendlichen. Falls Familien oder Elternteile einbezogen wurden, wird dies ausdrücklich angegeben; *k. A.* keine Angabe; *M* Mean; *SD* Standard deviation; *SDQ* Strengths and Difficulties Questionnaire; *SCARED* Screen for Child Anxiety Related Emotional Disorders; *CES-D* Center for Epidemiologic Studies Depression Scale; *PHQ-9* Patient Health Questionnaire; *GAD-7* Generalized Anxiety Disorder 7; *ISI* Insomnia Severity Index; *EAT-8* Eating Attitudes Test 8; *WHO-5* World Health Organization Well-Being Index 5

## Zur psychischen Situation von Kindern und Jugendlichen vor der Pandemie

Kinder und Jugendliche zählen in Bezug auf die Entwicklung von psychischen Auffälligkeiten zu einer der vulnerabelsten Gruppen. Laut Bundespsychotherapeutenkammer erkranken fast 20 % der Kinder und Jugendlichen innerhalb eines Jahres an einer psychischen Störung. Mehr als die Hälfte der psychischen Erkrankungen manifestieren sich bereits vor dem 19. Lebensjahr. Dabei zählen zu den häufigsten Erkrankungen Angststörungen, sowie depressive, hyperkinetische und dissoziale Störungen [3]. Bis zum Alter von 13 Jahren wurden durchgehend höhere Prävalenzen für psychische Störungen bei Jungen als bei Mädchen beobachtet. Im weiteren Verlauf der Adoleszenz erfolgten Angleichungen [17]. Insgesamt sind Jungen häufiger von externalisierenden Störungen betroffen. Mädchen leiden stärker unter Ess- und psychosozialen Störungen [16].

In einer repräsentativen Studie zur Gesundheit von Kindern und Jugendlichen in Deutschland (KiGGS) wurde gezeigt, dass bereits vor der Pandemie von 2003 bis 2006 19,9 % der Kinder psychisch auffällig waren [19]. In der zweiten KiGGS-Welle von 2014 bis 2017 lag die Prävalenz bei 16,9 %. Diese rückläufige Tendenz betrifft v. a. Jungen im Alter von 9 bis 17 Jahren. Mögliche Ursachen können hierfür sein, dass in den letzten Jahren zahlreiche Präventions- und Interventionsmaßnahmen initiiert wurden, die ihren Fokus auf die Förderung der psychischen Gesundheit von Kindern und Jugendlichen legten. Im Rahmen der KiGGS-Basiserhebung von 2003 bis 2006 konnte weiterhin gezeigt werden, dass 70 % der psychisch auffälligen Kinder und Jugendlichen keine psychotherapeutische Behandlung in Anspruch nahmen, möglicherweise aufgrund einer schwachen psychiatrisch-psychologischen Versorgungslage. In den letzten Jahren kam es jedoch zu einem Anstieg der Vertragsärztlichen Versorgung und damit auch zu einer stärkeren Inanspruchnahme von psychiatrisch-psychotherapeutischen Leistungen [19]. Laut dem Arztreport 2021 der BARMER hat sich die Zahl der Kinder und Jugendlichen, die sich in psychotherapeutischer Behandlung befinden, in den letzten Jahren verdoppelt. Demnach benötigten 823.000 Kinder und Jugendliche im Jahr 2019 psychotherapeutische Unterstützung und damit 104 % mehr als im Jahr 2009. Die häufigsten Behandlungsursachen 2019 waren laut Arztreport Reaktionen auf schwere Belastungen und Anpassungsstörungen sowie Depressionen [14].

## Die psychische Situation während der Coronapandemie

In einer repräsentativen Online-Befragung (*n* = 12.628) [20] sollte die Frage geklärt werden, wie Eltern und Kinder im Alter von 3 bis 15 Jahren die Coronakrise erleben und bewältigen. Zusätzlich wurde eine qualitative Interviewstudie durchgeführt. Dabei wurden Fragen zum Freizeitverhalten, zum Familienklima, zu Verhaltensproblemen und zur generellen Bewältigung der Coronasituation gestellt. Es zeigte sich, dass die Kinder aller Altersstufen deutlich häufiger das Internet nutzten (Kindergarten: 33 %; Grundschule 36 % und Sekundarstufe 72 %). Auch im Bereich des Familienklimas kam es zu wesentlichen Veränderungen: Überforderung, große finanzielle Belastungen oder Schwierigkeiten bei der Vereinbarkeit von Beruf und Familie führten zu Streitereien zwischen den Familienmitgliedern. 22 % der befragten Eltern gaben an, dass Konflikte und Chaos „häufig“ bzw. „sehr häufig“ Teil des Coronaalltags seien. Vor allem bei Familien, bei denen mehrere Kinder im Haushalt leben, war dies der Fall (25 % vs. 14 % bei Ein-Kind-Familien). 32 % der Eltern gaben an, dass die Ausgangs- und Kontaktbeschränkungen ihre Kinder stark belastete. Bei Eltern mit hohem Bildungsabschluss wurde häufiger angegeben, dass ihre Kinder die Situation „gut“ oder „sehr gut“ bewältigen ([Fach-]Hochschulabschluss: 72 %; Abitur: 65 %, mittlere Bildung: 55 %). Verhaltensprobleme, die bei den befragten Kindern festgestellt werden konnten, waren emotionale Schwierigkeiten (23 % auffällig; Vergleich Deutsche Normstichprobe [32]: 7,7 % auffällig) und Hyperaktivitätsprobleme (29 % auffällig; Vergleich Deutsche Normstichprobe [32]: 9,8 % auffällig). Vor allem Mädchen waren im Vergleich zu Jungen häufiger von emotionalen Schwierigkeiten betroffen (24 % vs. 21 %). Dagegen litten die Jungen stärker unter Hyperaktivitätsproblemen als Mädchen (31 % vs. 23 %).

Diese Ergebnisse konnten in der bundesweiten COPSY-Studie [27, 28] bestätigt werden. Die Daten wurden mit der BELLA-Kohortenstudie [26] verglichen, die in Deutschland vor der Pandemie durchgeführt wurde. Während der ersten Befragungswelle von Mai bis Juni 2020 gaben 69,4 % der teilnehmenden Kinder und Jugendlichen an, sich durch die coronabedingten Kontaktbeschränkungen belastet zu fühlen. In der zweiten Befragungswelle (*n* = 846) von Dezember 2020 bis Januar 2021 stieg dieser Wert weiter signifikant an (82,6 %; *p* < 0,001). Weiterhin berichtete ein Großteil der Teilnehmenden von deutlich weniger sozialen Kontakten als vor der Pandemie. Vor allem während der ersten Welle wurde dies signifikant häufiger angegeben als während der 2. Welle (Welle 1: 82,8 %; Welle 2: 76,1 %; *p* < 0,001). Mehr als ein Drittel der Befragten gab weiterhin an, dass sich durch die coronabedingten Kontaktbeschränkungen das Verhältnis zu ihren Freunden und Bekannten verschlechterte (Welle 1: 37,9 %; Welle 2 = 39,4 %). Etwa ein Viertel der Kinder und Jugendlichen stritten sich häufiger innerhalb der Familie (Welle 1 = 26,2 %; Welle 2 = 23,8 %).

Weiterhin berichtete die COPSY-Studie, dass sich die Lebensqualität der Kinder und Jugendlichen während der COVID-19-Pandemie signifikant verschlechterte. Vor der Pandemie gaben nur 15,3 % der Kinder und Jugendlichen an, von einer niedrigen Lebensqualität betroffen zu sein. Dagegen waren es in der ersten Welle bereits 40,2 % und in der 2. Welle sogar knapp die Hälfte der teilnehmenden Kinder und Jugendlichen (Welle 1 = 40,2 %; Welle 2 = 47,7 %; Prä-Pandemie = 15,3 %; *p* < 0,001).

Auch nahmen psychosomatische Beschwerden bei Kindern und Jugendlichen während der Pandemie deutlich zu. Vor allem zählten Gereiztheit, Schlafprobleme und Kopfschmerzen zu den häufigsten Symptomen (Gereiztheit: Prä-Pandemie: 39,8 %, Welle 2: 57,2 %; Schlafprobleme: Prä-Pandemie: 39,2 %, Welle 2: 47,7 %; Kopfschmerzen: Prä-Pandemie: 28,3 %, Welle 2: 46,4 %).

Die coronabedingten Kontaktbeschränkungen führten bei Kindern und Jugendlichen zu einem starken Gefühl der sozialen Isolation. Das Internet und insbesondere das Smartphone waren häufig die einzige Möglichkeit, mit Freunden in Kontakt zu bleiben und Informationen auszutauschen. Eine übertriebene Nutzung von digitalen Medien, in Form von Social Media und PC-Spielen, kann gesundheitsschädliche Auswirkungen haben [31]. So fand eine von der Deutschen Angestelltenkrankenkasse (DAK) in Auftrag gegebene Befragung zur Mediennutzung von Kindern und Jugendlichen heraus, dass die Nutzungsdauer von Computerspielen und sozialen Online-Medien von September 2019 bis April 2020 deutlich anstieg. Im September 2019 betrug die an Schultagen verbrachte Zeit 3 h und 15 min und stieg im April 2020 auf 5 h und 32 min. An Wochenenden und in den Ferien stieg die tägliche Nutzungsdauer sogar von 5 h 34 min im September 2019 auf 7 h 14 min im April 2020 [10, 11, 31].

In einer Studie von Pieh [23] wurde im Februar 2021 mit *n* = 3052 Jugendlichen der Zusammenhang zwischen dem Smartphone-Nutzungsverhalten und dem mentalen Wohlbefinden untersucht. Es zeigte sich, je öfter das Smartphone am Tag genutzt wurde, desto häufiger traten Depressionen, Angst‑, Ess- und Schlafstörungen auf.

Die COVID-19-Pandemie hat auch die psychiatrisch-psychologische Versorgungslage von Kindern und Jugendlichen weiter verschärft. In einer Umfrage der Deutschen Psychotherapeutenvereinigung (DPtV) unter 685 Kinder- und JugendlichenpsychotherapeutInnen wurde festgestellt, dass die Patientenanfragen im Januar 2021 im Vergleich zu Januar 2020 um 60,3 % gestiegen waren (Januar 2020: 3,7 Patientenanfragen pro Woche; Januar 2021: 5,9 Patientenanfragen pro Woche). Nur 34,5 % der Kinder und Jugendlichen konnte ein Erstgespräch angeboten werden, 11 % erhielten einen Behandlungsplatz innerhalb eines Monats. Fast 40 % mussten jedoch länger als 6 Monate warten [24].

## Risiko und protektive Faktoren

Stress, Ängste und ökonomische Krisen, bei einem gleichzeitigen Verlust möglicher Unterstützersysteme, spielen als Risikofaktoren während der Coronapandemie eine nicht zu unterschätzende Rolle [9]. Es gibt Anzeichen dafür, dass Kinder und Jugendliche, die bereits benachteiligt sind, auch die größte Gefährdung aufweisen, psychische Auffälligkeiten aufgrund der coronabedingten Einschränkungen zu entwickeln [8]. Ein hohes Risikopotential haben v. a. Kinder und Jugendliche mit einem niedrigen sozioökonomischen Status [5]. Familien mit einem höheren Einkommen haben die Möglichkeit, ihren Kindern Technologien zur Verfügung zu stellen, welche zum einen für schulische Aktivitäten (z. B. Homeschooling) und zum anderen für die Interaktion mit Gleichaltrigen genutzt werden können. Dadurch kann eine soziale Isolation aufgrund von Schulschließungen und Kontaktbeschränkungen abgemildert werden [7]. Auch die Wohnsituation kann eine deutliche Belastung darstellen. Leben die Familienmitglieder auf beengtem Raum (< 20 m^2^ Wohnfläche/Person) und haben keine eigenen Rückzugsmöglichkeiten, kann dies ebenfalls eine deutliche Beeinträchtigung für die psychische Gesundheit und die Lebensqualität darstellen [27, 28].

Jüngere Kinder, Kinder aus Familien mit einem Migrationshintergrund, Kinder und Jugendliche mit chronischen Erkrankungen, einem niedrigen Bildungsstatus der Eltern, partnerschaftliche Konflikte zwischen den Erziehenden oder auch psychische oder körperliche Erkrankungen bei Mutter oder Vater führen zu einer stärkeren Belastung [5, 26]. Für Kinder mit einer geistigen Behinderung kann es schwierig sein, die Situation und die Notwendigkeit der coronabedingten Einschränkungen zu verstehen. Dadurch können Gefühle wie Angst oder Aufregung zunehmen [8].

Ein wichtiger schützender Faktor vor psychischen Auffälligkeiten bei Kindern ist die Unterstützung ihrer Eltern. Eine offene Kommunikation zwischen Eltern und Kindern, über die Coronapandemie und ihre Auswirkungen, kann eine präventive Wirkung auf die mentale Gesundheit haben. Wichtig ist, sich die Sorgen und Ängste der Kinder anzuhören und einfühlsam darauf einzugehen. Eltern sollten authentisch über die eigenen Gefühle und Ängste sprechen. Durch die Normalisierung ihrer emotionalen Reaktion werden Kinder beruhigt und bestehende Ängste eingedämmt. Generell spielt eine stabile Eltern-Kind-Beziehung eine große Rolle als präventiver Faktor von psychischen Auffälligkeiten [6].

Aber auch persönliche Ressourcen, wie z. B. ein hohes Selbstwertgefühl, hohe Intelligenz, sowie ein offener und aktiver Charakter können protektive Faktoren sein [15].

Bestimmte digitale Anwendungen können in Maßen förderlich für die mentale Gesundheit von Kindern und Jugendlichen wirken. Gesundheitsdienste wie Telemedizin oder ein interaktiver Online-Unterricht sind in der Lage, die soziale Distanz zu überbrücken und die psychische und verhaltensbezogene Gesundheit von Kindern zu unterstützen [33].

**Abb. 1:**
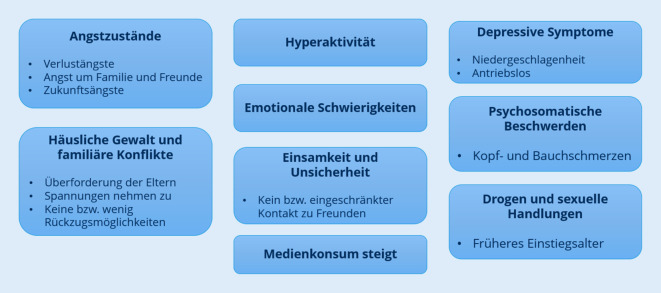
Mögliche negative Auswirkungen der Pandemie auf Kinder und Jugendliche

In Abb. 1 werden zusammenfassend die möglichen negativen Auswirkungen der Coronapandemie auf Kinder und Jugendliche gezeigt.

## Projektskizze: ProWell – digitale Schulungen für PädagogInnen zur Förderung der psychischen Gesundheit von Kindern und Jugendlichen bei Krisen der öffentlichen Gesundheit

In Deutschland gibt es eine Vielzahl von Projekten und Programmen die sich mit der Förderung des mentalen Wohlbefindens von Kindern und Jugendlichen beschäftigen. Allerdings bezieht sich nur ein kleiner Teil dieser Angebote auf die besonderen Belastungen der psychischen Gesundheit von SchülerInnen während der COVID-19-Pandemie. Mit Unterstützung durch das Erasmus+-Programm der EU startete 2021 das Projekt „ProWell – Protecting the mental wellbeing of our children during and after public health emergencies – digital training for teachers and educators“ (www.prowell-project.com). ProWell baut neben den hier dargestellten Ergebnissen wissenschaftlicher Studien auf eine umfangreiche Recherche zu bereits bestehenden nationalen und europaweiten Präventionsprojekten zur Förderung der psychischen Gesundheit von Kindern und Jugendlichen während Krisensituationen der öffentlichen Gesundheit. Zusätzlich wurden Fokusgruppen Interviews mit Lehrkräften und anderen PädagogInnen durchgeführt, um deren Vorstellungen und Anforderungen in der Entwicklung der Trainingsmodule zu berücksichtigen.

Durch digitale Schulungen von LehrerInnen und anderen PädagogInnen soll das psychische Wohlbefinden von Kindern und Jugendlichen während Krisen der öffentlichen Gesundheit gefördert werden. Die deutsche Projektleitung an der TU Dresden arbeitet mit Institutionen aus sechs Partnerländern zusammen (Griechenland, Zypern, Spanien, Italien, Frankreich und Kroatien). Gemeinsam wird ein modulares Schulungskonzept entwickelt, mit dem PädagogInnen die Möglichkeit erhalten, ihre Mental Health Literacy zu verbessern, psychische Auffälligkeiten bei ihren SchülerInnen leichter zu erkennen und mögliche Hilfsmaßnahmen rechtzeitig initiieren zu können. Dieses Schulungskonzept wird den pädagogischen Fachkräften auf einer frei verfügbaren E‑Learning Plattform zur Verfügung gestellt. Für eine Evaluation des Projekts, wird die Nutzung der Projekt-Webseite und der E‑Learning Plattform (u. a. Zahl der Nutzer und Downloads der verfügbaren Trainingsmaterialien) ausgewertet. Zusätzlich können Teilnehmende Feedback-Fragebögen ausfüllen, welche für die Weiterentwicklung des Projekts herangezogen werden. Das Projekt wird Anfang des Jahres 2023 fertiggestellt. Die geplanten Trainingsmodule von ProWell sind in Tab. 2 dargestellt.

**Tab. 2:** Geplante Trainingsmodule der E‑learning-Plattform von ProWell

Module 1	*Promoting communication skills between teachers and students/parents* (Förderung der Kommunikationsfähigkeiten zwischen LehrerInnen und SchülerInnen/Eltern)
Module 2	*Recognizing mental health difficulties of children and adolescents relevant to health emergencies & crisis situations* (Erkennen von psychischen Problemen bei Kindern und Jugendlichen während Krisen der öffentlichen Gesundheit)
Module 3	*Preventing & Coping with mental health difficulties of children/adolescents relevant to health emergency crisis* (Präventions- und Copingstrategien bei psychischen Problemen von Kindern und Jugendlichen während Krisen der öffentlichen Gesundheit)
Module 4	*Learning how to organize and implement mental health interventions at the school setting* (Interventionsstrategien zur Förderung der psychischen Gesundheit in der Schule)
Module 5	*Promoting teachers’ mental health and well-being* (Förderung der psychischen Gesundheit und des Wohlbefindens von LehrerInnen)
Module 6	*Digital literacy and the effect media had on mental health created by health emergency crisis* (Digitale Kompetenzen und die Auswirkungen der Medien auf die psychische Gesundheit während Krisen der öffentlichen Gesundheit)
Module 7	*General concepts of referral for mental health issues –* *including country specific information’s* (Unterstützungsangebote für Kinder und Jugendliche bei Problemen der psychischen Gesundheit – einschließlich länderspezifischer Informationen)

Mit den Trainingsmodulen werden dann auch bei zukünftigen Krisenereignissen im Bereich der öffentlichen Gesundheit effiziente Maßnahmen zur Förderung der psychischen Gesundheit von Kindern und Jugendlichen bereitstehen, die von PädagogInnen und anderen in der Erziehungsarbeit tätigen Personen angewandt werden können.

## Fazit für die Praxis

- Die psychische Belastung von Kindern und Jugendlichen hat während der Coronapandemie deutlich zugenommen. Ursächlich hierfür sind v. a. die Ausgangs- und Kontaktbeschränkungen, Ängste und Unsicherheiten bezüglich des Coronavirus, aber auch familiäre Spannungen und Konflikte.
- Zu den häufigsten Auffälligkeiten zählen depressive Symptome, Angstzustände, Einsamkeit und psychosomatische Beschwerden, wie Kopf- und Bauchschmerzen, Gereiztheit, Konzentrationsschwierigkeiten oder Schlafstörungen.
- Dringend benötigte Therapieplätze sind rar und mit langen Wartezeiten verbunden.
- ProWell ist ein digitales Schulungsprogramm für LehrerInnen und andere PädagogInnen zur Stärkung der psychischen Gesundheit und des mentalen Wohlbefindens von SchülerInnen während Krisen der öffentlichen Gesundheit.
